# Supplementary material for: Microstructured Polymeric Fabrics Modulating the Paracrine Activity of Adipose-Derived Stem Cells
Source: Int J Mol Sci. 2023 Jun 14;24(12):10123. doi: 10.3390/ijms241210123 (PMC10299066; doi:10.3390/ijms241210123)
Supplement: Supplementary file 1 [file ijms-24-10123-s001.zip › ijms-2378189-supplementary.pdf]

Article

Microstructured Polymeric Fabrics Modulating the Paracrine Activity of Adipose-Derived Stem Cells

Federica Grilli [1,2], Ennio Albanesi [3], Beatriz Pelacho [4,5], Felipe Prosper [4,5], Paolo Decuzzi [1,†] and Daniele Di Mascolo [1,6,*,†]

[1]Laboratory of Nanotechnology for Precision Medicine, Fondazione Istituto Italiano di Tecnologia, Via Morego 30, Genoa 16163, Italy

[2]Department of Informatics, Bioengineering, Robotics, and Systems Engineering (DIBRIS),University of Genoa, 16145 Genoa, Italy

[3]Department of Neuroscience and Brain Technologies, Fondazione Istituto Italiano di Tecnologia, Via Morego 30, Genoa 16163, Italy

[4]Laboratory of Regenerative Medicine, Center for Applied Medical Research - University of Navarra,Av.DaPio XII, 55, 31008,Pamplona, Spain

[5]Instituto de Investigación Sanitaria de Navarra (IdiSNA),31008 Pamplona, Spain

[6] Department of Electrical and Information Engineering, Politecnico di Bari, 70126, Bari, Italy

†These authors share the senior authorship

* Correspondence: Daniele Di Mascolo, PhD – daniele.dimascolo@iit.it

SUPPORTING INFORMATION

Numerosity

	Tukey's multiple comparisons test	p-value
24h	5x5 vs 10x10	0.1331
	5x5 vs 20x20	0.2040
	10x10 vs 20x20	0.8872
48h	5x5 vs 10x10	0.4994
	5x5 vs 20x20	0.9452
	10x10 vs 20x20	0.6328
72h	5x5 vs 10x10	0.9454
	5x5 vs 20x20	0.9076
	10x10 vs 20x20	0.7348

Perimeter

	Tukey's multiple comparisons test	p-value
24h	5x5 vs 10x10	0.0356 (*)
	5x5 vs 20x20	<0.0001 (****)
	10x10 vs 20x20	<0.0001 (****)
48h	5x5 vs 10x10	0.9613
	5x5 vs 20x20	<0.0001 (****)
	10x10 vs 20x20	<0.0001 (****)
72h	5x5 vs 10x10	0.0382 (*)
	5x5 vs 20x20	<0.0001 (****)
	10x10 vs 20x20	<0.0001 (****)

Aspect Ratio

	Tukey's multiple comparisons test	p-value
24h	5x5 vs 10x10	0.0001 (**)
	5x5 vs 20x20	<0.0001 (****)
	10x10 vs 20x20	0.5820
48h	5x5 vs 10x10	<0.0001 (****)
	5x5 vs 20x20	0.0790
	10x10 vs 20x20	0.0008 (**)
72h	5x5 vs 10x10	0.2256
	5x5 vs 20x20	0.9919
	10x10 vs 20x20	0.1310

Comparison among the the three PLGA fabrics at 24h

Comparison among the the three PLGA fabrics at 48h

Comparison among the the three PLGA fabrics at 72h

Supporting Figure S1. Statistical Analysis for the stem cell spheroid growth as a function of time and for three different PLGA fabrics. Differences are considered statistically significant when $p<0.05$ (*), $p<0.01$ (**), and $p<0.0001$ (****).

2D

	Tukey's multiple comparisons test	p-value
LIVE	24h vs 48h	0.2742
	24h vs 72h	0.1675
	48h vs 72h	0.9407
APOPTOTIC	24h vs 48h	0.2399
	24h vs 72h	0.1432
	48h vs 72h	0.9387
DEAD	24h vs 48h	0.8383
	24h vs 72h	0.1539
	48h vs 72h	0.3498

2D collagen

	Tukey's multiple comparisons test	p-value
LIVE	24h vs 48h	0.7550
	24h vs 72h	0.0322 (*)
	48h vs 72h	0.1221
APOPTOTIC	24h vs 48h	0.8354
	24h vs 72h	0.0895
	48h vs 72h	0.0304 (*)
DEAD	24h vs 48h	0.0011 (**)
	24h vs 72h	0.1598
	48h vs 72h	0.0507

5x5μm PLGA fabric

	Tukey's multiple comparisons test	p-value
LIVE	24h vs 48h	0.9433
	24h vs 72h	0.9859
	48h vs 72h	0.8986
APOPTOTIC	24h vs 48h	0.9606
	24h vs 72h	0.9951
	48h vs 72h	0.9427
DEAD	24h vs 48h	0.8178
	24h vs 72h	0.7942
	48h vs 72h	0.4889

10x10μm PLGA fabric

	Tukey's multiple comparisons test	p-value
LIVE	24h vs 48h	0.8631
	24h vs 72h	0.7167
	48h vs 72h	0.9611
APOPTOTIC	24h vs 48h	0.9533
	24h vs 72h	0.6794
	48h vs 72h	0.8452
DEAD	24h vs 48h	0.6307
	24h vs 72h	>0.9999
	48h vs 72h	0.6242

20x20μm PLGA fabric

	Tukey's multiple comparisons test	p-value
LIVE	24h vs 48h	0.8309
	24h vs 72h	0.7608
	48h vs 72h	0.9918
APOPTOTIC	24h vs 48h	0.7737
	24h vs 72h	0.8457
	48h vs 72h	0.9910
DEAD	24h vs 48h	0.5590
	24h vs 72h	0.7943
	48h vs 72h	0.2802

% of live cells over time on different substrates

% of apoptotic cells over time on different substrates

% of dead cells over time on different substrates

Supporting Figure S2. Statistical Analysis for the stem cell spheroid viability as a function of time and for three different PLGA fabrics. Differences were considered statistically significant for $p<0.05$ (*) and $p<0.01$ (**).
